# Approach of Heterogeneous Spectrum Involving 3beta-Hydroxysteroid Dehydrogenase 2 Deficiency

**DOI:** 10.3390/diagnostics12092168

**Published:** 2022-09-07

**Authors:** Andreea Gabriela Nicola, Mara Carsote, Ana-Maria Gheorghe, Eugenia Petrova, Alexandru Dan Popescu, Adela Nicoleta Staicu, Mihaela Jana Țuculină, Cristian Petcu, Ionela Teodora Dascălu, Tiberiu Tircă

**Affiliations:** 1 Department of Oro-Dental Prevention, Faculty of Dental Medicine, University of Medicine and Pharmacy of Craiova, 200349 Craiova, Romania; 2 Department of Endocrinology, Carol Davila University of Medicine and Pharmacy, 011863 Bucharest, Romania; 3 Department of Endocrinology, C.I. Parhon National Institute of Endocrinology, Aviatorilor Ave 34-38, Sector 1, 011863 Bucharest, Romania; 4 Department of Endodontics, Faculty of Dental Medicine, University of Medicine and Pharmacy of Craiova, 200349 Craiova, Romania; 5 Department of Orthodontics, Faculty of Dental Medicine, University of Medicine and Pharmacy of Craiova, 200349 Craiova, Romania

**Keywords:** 3-beta-hydroxysteroid dehydrogenase, congenital adrenal hyperplasia, hirsutism, androgen, gene, salt-wasting, enzyme, disorder of sexual development, TART, OART

## Abstract

We aim to review data on 3beta-hydroxysteroid dehydrogenase type II (3βHSD2) deficiency. We identified 30 studies within the last decade on PubMed: 1 longitudinal study (N = 14), 2 cross-sectional studies, 1 retrospective study (N = 16), and 26 case reports (total: 98 individuals). Regarding geographic area: Algeria (N = 14), Turkey (N = 31), China (2 case reports), Morocco (2 sisters), Anatolia (6 cases), and Italy (N = 1). Patients’ age varied from first days of life to puberty; the oldest was of 34 y. Majority forms displayed were salt-wasting (SW); some associated disorders of sexual development (DSD) were attendant also—mostly 46,XY males and mild virilisation in some 46,XX females. SW pushed forward an early diagnosis due to severity of SW crisis. The clinical spectrum goes to: premature puberty (80%); 9 with testicular adrenal rest tumours (TARTs); one female with ovarian adrenal rest tumours (OARTs), and some cases with adrenal hyperplasia; cardio-metabolic complications, including iatrogenic Cushing’ syndrome. More incidental (unusual) associations include: 1 subject with Barter syndrome, 1 Addison’s disease, 2 subjects of Klinefelter syndrome (47,XXY/46,XX, respective 47,XXY). Neonatal screening for 21OHD was the scenario of detection in some cases; 17OHP might be elevated due to peripheral production (pitfall for misdiagnosis of 21OHD). An ACTH stimulation test was used in 2 studies. Liquid chromatography tandem–mass spectrometry unequivocally sustains the diagnostic by expressing high baseline 17OH-pregnenolone to cortisol ratio as well as 11-oxyandrogen levels. *HSD3B2* gene sequencing was provided in 26 articles; around 20 mutations were described as “novel pathogenic mutation” (frameshift, missense or nonsense); many subjects had a consanguineous background. The current COVID-19 pandemic showed that CAH-associated chronic adrenal insufficiency is at higher risk. Non-adherence to hormonal replacement contributed to TARTs growth, thus making them surgery candidates. To our knowledge, this is the largest study on published cases strictly concerning 3βHSD2 deficiency according to our methodology. Adequate case management underlines the recent shift from evidence-based medicine to individualized (patient-oriented) medicine, this approach being particularly applicable in this exceptional and challenging disorder.

## 1. Introduction

Congenital adrenal hyperplasia (CAH) represents a group of disorders affecting adrenal and extra-adrenal steroidogenesis, caused by a deficiency in at least one of the enzymes involved in the synthesis of cortisol from cholesterol at the level of the adrenal cortex [1,2,3]. The 21-hydroxylase (21OH) deficiency underling *CYP21A2* gene mutations on chromosome 6p21.22 is the most frequent form among all seven types of enzymes deficiencies, representing 90–95% all CAH cases [2,4,5,6]. Compared to a large number of studies addressing this particular type of CAH, little is published concerning other forms, particularly 3beta-hydroxysteroid dehydrogenase type II (3βHSD2) deficiency [2,4,5,6].

Regarding the epidemiologic impact, 21OH deficiency is followed by 17-hydroxylase/17,20-lyase deficiency in Brazil or by 11-beta hydroxylase deficiency in the Middle Est (mostly depending on geographic region and ethnic group) [2,4]. Worldwide, 3βHSD2 deficiency is among the rarest of all types, with a prevalence < 1 case/1,000,000 births, except for some specific populations (like the Amish community in Lancaster County, PA, USA) [2,4].

Since therapy with adrenocortical hormones for CAH was introduced by mid-20th century, a massive progress was registered; however, to this day, CAH is still a challenging condition, in terms of recognition and lifelong management [7,8,9]. Disease-associated burdens include hormonal anomalies–related comorbidities as well as side effects of the long-term therapy, varying from life-threatening acute adrenal insufficiency (with a mortality rate of 0.5 per 100 patient years in the absence of specific treatment) to impairment of growth, puberty, in addition to cardio-metabolic, skeletal and reproductive issues [7,10,11,12,13,14].

Lately, massive progress has been established in terms of molecular and genetic diagnosis, mostly considering 21OH deficiency [15]. Moreover, traditional regimes of adrenal hormone replacement are under evaluation in order to be reconsidered in terms of using alternative hydrocortisone doses with modified pharmacokinetics to mimic circadian rhythm or formulas with prolonged release, or in terms of new drugs like inhibitors of androgen biosynthesis, inhibitors of ACTH (Adrenocorticotropic Hormone) secretion through CRF (Corticotrophin Releasing Factor) type 1 receptor antagonists, and, hopefully, gene therapy [16,17,18,19].

3βHSD2 deficiency presents a heterogeneous clinical spectrum, meaning signs and symptoms of adrenal insufficiency, with or without salt wasting (SW), and different degrees of DSD (disorders of sexual development), especially in 46,XY patients [20]. The severity of this spectrum varies from life-threatening situations when adequate recognition is mandatory for patients’ survival, especially at very young ages, to mild forms that are diagnosed during puberty, especially in females who might be considered first as having polycystic ovaries syndrome (POS) [21].

We aim to overview latest published data regarding the most uncommon form of CAH, namely 3βHSD2 deficiency in order to provide an update on terms of clinical, hormonal and genetic assessment, and management.

## 2. Methods

This is a narrative review of the English-published papers regarding 3βHSD2 deficiency through a PubMed-based search using the following keywords: “3beta-hydroxysteroid dehydrogenase deficiency” and/or “*HSD3B2*” in different associations with “adrenal”, “testes”, “ovaries”, “salt wasting”, “hirsutism” “menses”, “androgens”, and “congenital adrenal hyperplasia”. The inclusion criteria were clinically, hormonally, and genetically relevant (original) studies on 3βHSD2 deficiency within the last decade (between January 2012 and August 2022) regardless of the level of statistical evidence.

In terms of what statistical evidence is concerned, we identified 30 original articles which included: 1 longitudinal study (N = 14), 2 cross-sectional studies (N1 = 31, N2 = 386 of CAHs with 6 cases underling 3βHSD deficiency), 1 retrospective study (N = 16), and 26 case reports (of either 1, 2 or 3 patients), for a total of 98 individuals. The largest cohorts involving only this type of enzyme defect were of 31, 16, and 14 individuals. Regarding geographic area, we mention Algeria (N = 14), Turkey (N = 31), China (2 case reports), Morocco (2 sisters), Anatolia (6 cases), and Italy (one case). Patients’ age varied from first days of life to puberty; the oldest patient was 34 years’ old. Genetic analysis was specifically provided for *HSD3B2* gene in 26 papers. We integrated these latest studies concerning 3βHSD2 deficiency into the general approach of CAH.

### 2.1. 3βHSD2 Deficiency: General Frame of Approach

3βHSD2 deficiency, the rarest form of CAH, is part of the non-21OH-related CAH cluster which overall counts for less than 5–10% of CAHs (generally, 5–8% for 11-beta hydroxylase deficiency, and less than 1% for 17-hydroxylase deficiency in association with 3βHSD2 deficiency) [22,23]. As mentioned, some exceptions of a relatively higher incidence are registered in areas like Old Order Amish of North America due to a *c.35GA* founder mutation [22,24,25,26].

There are two types of CAH underling 3βHSD2 deficiency: SW due to associated aldosterone deficiency, and non-SW that involves a normal mineralocorticoids production. DSDs are positive in any of these forms, while non-SW also includes a milder presentation which may be predominantly expressed as hirsutism and menstrual cycle anomalies [27]. Other authors consider that we should discuss of 3 distinct forms: SW, non-SW, and non-classical type as a different entity with pubertal onset especially in females [27,28]. Generally, 95% of cases diagnosed with paediatric adrenal insufficiency are caused by CAH, but 3βHSD2 deficiency has a modest epidemiological impact opposite to 21OH deficiency [29]. In teenagers and young adults, the most frequent cause of adrenal insufficiency is Addison’s disease followed by CAH, overall chronic adrenal failure having more than 30 aetiologies [30].

3βHSD is an enzyme that converts ∆5-steroids: pregnenolone (Preg), 17-hydroxypregnenolone (17OH-Preg), dehydroepiandrosterone (DHEA), and androstenediol to the corresponding ∆4-steroids, meaning progesterone, 17-hydroxyprogesterone (17OHP), androstenedione, and testosterone [5].

In the human genome, there are two active 3βHSD genes located on chromosome 1p13.1; these encode two 3βHSD isoenzymes [5,31,32]. The 3βHSD type 1 isoenzyme is found in several tissues such as placenta, brain, liver, and breast, skin, and prostate while the type II enzyme is identified at the level of adrenals and gonads [5,31,32,33]. In contrast with the type II isoenzyme, which acts only on intra-glandular steroids, 3βHSD type 1 is able to act on low levels of circulating steroids, due to its low Michaelis–Menten constant [5,31,32,33]. For instance, this enables the liver isoenzyme to convert some of the elevated 17OH-Preg into 17OHP in cases with 3βHSD type II deficiency [5,31,32,34]. There have not been reported mutations of the 3βHSD type I gene, presumably because such mutation would prevent progesterone synthesis in the placenta, leading to spontaneous abortion [33,34].

The advance of molecular and genetic diagnosis is mostly useful in different types of CAH, starting with the most frequent “mutation”, namely 21OH deficiency, since a level of genotype–phenotype correlation is expected. However, in 3βHSD2 deficiency the current level of knowledge makes this association less predictable due to the paucity of studies [29].

The most significant hormonal changes associated with 3βHSD deficiency are high ratios of the Δ5-steroids (Preg, 17OH-Preg, DHEA, and androstenediol) to corresponding Δ4-steroids (progesterone, 17OHP, androstenedione, and testosterone), especially high 17OH-Preg/17OHP, elevated serum levels of 17OH-Preg, DHEA and dehydroepiandrosterone sulfate (DHEA-S) and increased urinary levels of their corresponding metabolites [5,35,36].

A useful diagnostic (dynamic) test is the ACTH stimulation test: 17OH-Preg, cortisol, 17OHP, DHEA-S, and androstenedione are measured before and one hour after administration of 250 µg of synthetic ACTH (Cosyntropin) [35]. In patients with 3βHSD2 deficiency, 17OH-Preg is expected to be >150 nmol/L after stimulation, while 17OH-Preg to 17OHP ratio is expected to become very high [37].

In the absence of treatment, SW forms, manifesting through vomiting and dehydration, may cause hypoglycemia, hyperkalemic acidosis, and hemodynamic instability including death [37]. Severe 3βHSD2 deficiency may lead to disorders of sex development (DSD) with ambiguous genitalia at birth (46,XY or 46,XX individuals); DSDs may accompany SW or non-SW type. Milder forms display females with virilization in association with menstrual cycle anomalies, while males have incomplete masculinization (non-classical phenotype) [37].

Management of 3βHSD2 deficiency is similar to other types of CAH, with glucocorticoid and mineralocorticoid replacement therapy in cases with severe enzyme deficiency. Concerning the paediatric population, treatment with hydrocortisone 10–15 mg/m^2^/day is preferred, while in adulthood long-acting glucocorticoids can be used. Mineralocorticoid replacement consists of therapy with fludrocortisone 0.1 mg/day, adjusted depending on plasma renin activity levels. Females with androgen excess (non-classical type) are candidates for oral contraceptives and anti-androgenic drugs in cases with severe hirsutism and menstrual cycle disturbances [35].

### 2.2. Clinical Presentation of Patients Confirmed with 3βHSD Type II Deficiency

We synthetized the studies published according to the mentioned methodology in Table 1 [5,22,25,26,27,31,38,39,40,41,42,43,44,45,46,47,48,49,50,51,52,53,54,55,56,57,58,59,60,61].

Most articles introduce cases with SW forms, which seem like a common presentation due to its severity. The majority of patients were diagnosed after a SW crisis (vomiting, diarrhoea, hyponatremia, hyperkaliaemia, and hypoglycaemia) early during childhood or soon after birth [22,25,26,31,38,40,41,43,44,47,50,53,55,57,58]. Other signs and symptoms that are described under these circumstances are a lack of sweating, malnutrition, and developmental delay [31,50]. Some patients with SW associated DSD with males (mostly 46,XY males and mild virilisation in some 46,XX females; these generally are less affected concerning the spectrum of DSDs). Most cases of ambiguous genitalia were found in male patients who were affected by undermasculinization and presented with hypospadias, embedded penis, micropenis, scrotum bifidum, cryptorchidism, and vaginal introitus [22,25,26,31,38,39,40,43,44,46,47,48,49,53,56,57,58,59]. Female patients had either normal genitalia or were mildly virilised (for instance, clitoromegaly) [22,25,26,31,38,40,43,44,46,47,48,49,53,56,57,58,59,61]. Other elements of clinical spectrum include: premature pubarche, gynecomastia, obesity, intellectual and developmental delay, and neurological sequelae (irritability, convulsions, etc. [22,31,38,46,47,51,57,61]. We mention the study of Ladjouze A. et al., in 14 Algerian patients with SW type who associated a median Prader stage of 2 for females (N = 8/14) at median age of 20 days without posterior labial fusion, while males had a median external masculinization score of 6 (N = 6/14). During follow-up, the same population showed, according to the Tanner system: premature pubarche (N = 4/14; female/male ratio of 3/1), spontaneous puberty (N = 6/14, female/male ratio of 5/1) at the age of 11 y respective for boys, respective 11.5 y for girls; overall 4/8 females reached menarche by the age of 14.3 y. A median IQ (intelligence quotient) of 90 was identified (2/14 patients had an IQ > 100 and 3/14 of studied individuals with an IQ < 70) [38].

The largest study on 3βHSD2 deficiency was a cross-sectional, nine-centred, Turkish cohort on 31 patients (average age of 6.6 ± 5.1 y, female/male ratio of 12/19). The authors identified SW and non-SW forms with a heterogeneous spectrum: homozygous pathogenic missense mutations of >5% wild type-associated non-SW phenotype; all males (19/31) had ambiguous genitalia at birth, while one female (1/12) had virilised genitalia. Premature puberty was confirmed in 80% of cases, despite the subjects of the study being on hydrocortisone therapy; through teenager years, females displayed irregular menstrual cycles, ultrasound aspects of polycystic ovaries, while males had hypogonadism and TARTs (2 brothers) [26]. Overall, the researchers concluded that intact mineralocorticoid production and unvirilized genitalia in 46,XX females represent the major pitfalls of missing the diagnosis of 3βHSD2 deficiency, while the particular constellation of an androgens profile might be attributed to other forms of CAH [26].

### 2.3. Biochemical/Hormonal Assessments in 3βHSD Type II Deficiency

Most relevant endocrine assays are displayed in Table 2 (some of the patients were undergoing hormonal replacement) [22,25,26,27,31,38,39,40,41,43,45,46,47,48,49,50,51,52,53,54,55,56,57,58,59].

The diagnosis of 3βHSD2 deficiency was established during neonatal screening for 21OH deficiency due to increased 17OHP levels [22,38,52,54,58]. High-risk molecular screening for patients with a family history of CAH was also performed in some patients [22,38].

In terms of overall hormonal picture, as expected, patients had high levels of 17OH-Preg, ACTH, DHEA, DHEA-S, 17OHP, renin, testosterone, androstenedione, and elevated ratios of 17OH-Preg to 17OHP ratio, respective of the 17OH-Preg to cortisol ratio [62]. The ACTH stimulation test was performed in 2 studies and showed an increase of 17OH-Preg (to a maximum of 1979 ng/dL), as well as a high 17OH-Preg to 17OHP ratio of 35.3 [48,51].

Ladjouze A. et al., showed that, unless the patients were under glucocorticoid replacement therapy, 17OHP levels were above upper limit in each patient. A value of 17OH-Preg above 90 nmol/L was found to be suggestive for the diagnosis of 3βHSD2 deficiency (a median of 139.7 nmol/L). DHEA-S revealed a wide area of values, between 9.4 and 5441.3 nmol/L (a median of 501.2 nmol/L). ACTH was abnormally high in 4/14 patients depending on the severity of genetic defect and the disease control achieved through daily hydrocortisone [38].

Li Z. et al., published a 5-case Chinese study on CAH (non-21OHD deficiency) and identified a 9-year-old boy with 3βHSD2 deficiency. Liquid chromatography tandem–mass spectrometry (LC-MS/MS) confirmed high levels of 17OHP and testosterone and very high values of DHEAS and androstenedione. High testosterone caused an enlargement of the testes within the last 5 years of surveillance in addition to accelerated height growth (+1.36 SD for his age), while his older brother sharing the same genetic defect had a final adult height deficiency of −3.71 SD [27]. Also, Guran T. et al. performed an LC-MS/MS analysis in 19/31 patients with 3βHSD2 deficiency; this showed an improvement of hormonal panel under adequate adrenocortical hormonal therapy; subjects who were 8 years and older showed higher levels of DHEAS and testosterone than patients below this cut off of age [26]. LC-MS/MS unequivocally sustained the diagnosis based on increased baseline 17OH-Preg to cortisol ratio as well as 11-oxyandrogen levels [26].

As mentioned by Fanis P. et al., the “pivotal role” of using gas chromatography-mass spectrometry in order to perform the urinary assays concerning steroids metabolomics finds its place in 3βHSD2 deficiency. In this case, the method revealed a rather late diagnosis of CAH, an 8.5-year-old boy (46,XY) with precocious puberty due to high testosterone (a 4-y advance of bone age versus his biological age) who was born with ambiguous genitalia and associated neonatal SW crisis, followed by gynecomastia, adrenarche and relapse of SW—related clinical elements by the age of 3.5 y [40].

Alternatively, liquid chromatography/electrospray ionization tandem mass spectrometry was used to assess urinary panel of cholesterol metabolites as a potential metabolomics approach to 3βHSD2 deficiency [63].

### 2.4. Genetic Background: HSD3B2

*HSD3B2* gene sequencing was provided in 26 articles; around 20 mutations were described as a “novel pathogenic mutation” [5,22,25,26,27,31,38,39,40,41,43,44,45,46,47,48,49,50,52,53,54,57,58,59,60,61] (Table 3).

There are over 40 pathogenic variants of the *HSD3B2* gene [64]. Loss of function mutations such as frameshift, in-frame deletions and nonsense mutations are associated with SW, while missense mutations generally lead to non-SW and non-classical forms of the disease [24,64]. Homozygous status leads to a more severe clinical manifestation; many of these patients had a parental history of consanguinity with parents often being first- or second- degree cousins [25,38,40,46,48,50,52,57]. This can be attributed to the fact that consanguinity may increase the risk of any autosomal recessive diseases, including 3βHSD2 deficiency [38,65]. We prior mentioned a single centric longitudinal study from Algeria which was published in 2022 on 14 patients (female/male ratio of 8/6), between 2007 and 2021. The subjects came from 10 families, and 8 of them were consanguineous, all individuals displaying p.Pro222Gln mutation (based on Sanger sequencing), except for two sisters with a novel mutation—a deletion of 3βHSD2 gene at the level of c.453_464del or p. (Thr152_Pro155del). Ladjouze A. et al., performed this genetic analysis at a median age of 20 days (between 0 and 390 days) while all subjects presented SW form (N = 14/14) in addition to DSD in majority cases (N = 10/14) [38].

Sanger sequencing also showed 11 homozygous pathogenic mutations that involved 6 novel mutations, including a c0.967A > G (p.N323D) which was positive in 14/31 patients coming from 11 Turkish families (Black Sea area), and homozygous c0.652T > C (p.S218P) mutation in 4 subjects originating from 4 families of Anatolian origin [26]. Another two studies presented patients from non-consanguineous families, with CAH due to 3βHSD2 deficiency caused by uniparental isodisomy [41,49]. As seen in other disorders, this is a condition characterised by two homologous chromosomes or regions which are inherited from the same parent [66].

Several case reports provided gene sequencing, as well. For instance, c.674T > A (p.V225D) mutation was confirmed on a 9-year-old Chinese boy with maternal and paternal inheritance of the genetic defect (the condition affected his older brother, too) [27]. Yu L. et al. described two novel mutations on a male subject: one with paternal inheritance—C.776 C > T—and the other from his mother—C.674 T > A —which might be responsible for the presence of bilateral TARTs [39]. Fanis P. et al., revealed a novel nonsense mutation—p.Lys36Ter (homozygosity, consanguineous parents)—in addition to a heterozygous status for *CYP21A2* gene—p.Val281Leu [40]. Teasdale SL. and Morton A. identified a novel mutation on a 46,XY subject with ambiguous genitalia who was first considered as having androgen insensitivity syndrome and became clinically relevant as CAH after the onset of the adrenarche 931C > T(p.Gln311) variant in addition to a previously diagnosed c.244G > A (p.Ala82Thr) mutation [47]. In 2017, a novel mutation of homozygous type was reported—p.W355R (c.763 T > C)—on two brothers who presented TARTs diagnosed during the first, respective the fourth year of life [48]. A 46,XX female was confirmed with a novel nonsense homozygous mutation Q334X of *HSD3B2* gene; the subject was born without DSD, but presented an SW crisis at the age of 13 days; 17OHP screening assays were positive, and, despite initially being considered as having 21OH deficiency, *CYP21A2* gene showed no mutations, thus the patient was re-assessed [60]. One series of 3 cases identified 2 novel pathogenic variants c.154_162delinsTCCTGTT and c.674T > A [31]. The first mutation in Italy was described in 2016 on a 46,XY boy coming from a non-consanguineous family admitted for DSD and SW: a novel homozygous frameshift mutation—a single nucleotide deletion at codon 319 (GTC(Val)x2192;GC) [53]. Of note, this patient who presented perineal hypospadias and microphallus in association with biochemistry anomalies due to SW crisis like hyponatremia was also included in a larger study concerning acute manifestations due to aldosterone deficiency (but we registered only the first report) [67]. Also, a relatively large study on different types of CAH identified 6 cases of 3βHSD2 deficiency introducing 4 novel mutations in Anatolian population [44].

### 2.5. Fertility Issues

Data regarding fertility are limited. Spermogram was analysed in several articles [25,45,48]. One case report showed a normal sperm count and percentage of sperm with typical morphology, but subnormal vitality of 41% (normal > 58%) in a 24-year-old male diagnosed with a SW form due to a 3βHSD2 deficiency caused by a c.687del27 homozygous mutation, as revealed by gene sequencing. The 46,XY male patient experienced neonatal SW crisis in addition to micropenis, hypospadias and bilateral intra-scrotal testicles, followed by normal puberty by the age of 15 years and current apparently optimal fertility potential, as opposite to majority of the other published data [25]. Another case showed an unlikely fertile male with 3βHSD2 deficiency due to a c.687del27 deletion, whose testis histology in late puberty revealed few tubules with arrested spermatogenesis. He showed no neoplastic changes and was therefore considered low risk for malignancy [57]. Another two case reports showed azoospermia; however, in these cases genetic testing was not performed [45,56].

Fertility in people affected by CAH is influenced by the severity of the condition, with most cases of infertility occurring in people with SW forms [68]. Generally, infertility in CAH appears to be connected with multiple factors, such as ambiguous genitalia and virilization of the external genitalia in females, excessive androgen secretion leading to impaired hypothalamic-pituitary-ovarian axis function, and various psychosocial factors [68,69].

We generally know that females with non-severe CAH might display anomalies of menstrual cycle and rarely conceive, but the level of statistical evidence remains very low regarding 3βHSD2 deficiency, and no particular study specifically addressed the fertility profile in females with non-classical form according to our mentioned methodology.

Concerning pre-implantation diagnosis and an associated particular approach, we mention a case study that introduced a healthy boy born via in vitro fertilization after pre-implantation diagnosis and identification of a healthy heterozygote. The child’s parents had heterogeneous mutations causing 3βHSD deficiency (c.690G > A) and a history of affected children [42].

### 2.6. Complications & Outcome

3βHSD3 deficiency may cause a heterogonous picture of complications: life threatening acute adrenal insufficiency manifested as adrenal crisis, hypoglycemia, and hyperkalemic acidosis; failure to thrive or advanced skeletal maturation; neurological impairments following perinatal asphyxia, etc. [22,55,57]. Mild non-SW forms might be misdiagnosed as having POS due to hirsutism, acne, and menstrual cycle anomalies (non-classical CAH) [22,38]. Ultrasound and computed tomography sometimes show adrenal hyperplasia [38,50]. Orchialgia and testicular masses were described in patients with testicular adrenal rest tumours (TARTs) [22,38,39,48,56].

Associated tumours like ovarian adrenal rest tumours (OART), respective TARTs (ectopic adrenal tissue at testes) were identified in 9 patients [22,38,39,45,48,56].

Genetic mutations associated with TARTs were c.35G > A, Cys-72-Arg in homozygote form or as compound heterozygous with deletion, c.674T > A, c.776 C > T, p.W355R (c.763 T > C), p.(Pro222Gln) [22,26,38,39,45,48,57]. Non-malignant Sertoli-only cell tumour (which is not part of TARTs) was associated with a homozygote c.687del27 of deletion type on a 46,XY male with ambiguous genitalia (SW form). He also proved at puberty some signs of virilisation due to testes and peripheral sources of testosterone as well as breast enlargement and high oestrogens levels, probably through peripheral production [57]. The first adult report of TARTs with extra-testicular (perirenal) location was introduced in 2012 fn a 23-year-old male with suboptimal replacement therapy (homozygous 694C > G mutation within the *HSD3B2* gene) [5].

The distinction between TART and Leydig cell tumours is sometimes difficult. We mention the case of a 34-year-old adult who, despite early treatment with glucocorticoids and mineralocorticoids (the first SW crisis was at the age of 1 month), was diagnosed with bilateral testicular lumps by the age of 13. An open testicular biopsy raised the question of a malignancy, and due to the tumour-related local accuses (masses of 4 cm) and azoospermia, bilateral orchiectomy was decided upon and later confirmed TARTs [45]. Interestingly, increased DHEAS seemed resistant to adequate adrenocortical hormonal replacement before surgery, while testosterone was within normal limits; thus, the decision of radical intervention versus testes-sparing surgery is even more challenging [45]. Another case of a 33-year-old male presented contra-lateral TARTs after a history of surgery for apparently a Leydig cell tumour of the testes. However, adrenal insufficiency due to a mild form of CAH was eventually recognized and both tumours were reconsidered as TARTs [56]. Testicular biopsy may be performed at young ages; this might add value regarding CAH evolution and hormonal control, for instance, to take into consideration the switch to another glucocorticoid formula or regime. We mention two siblings of 3.5 y and, respective, of 22 months who were diagnosed with TARTs through a testes biopsy; dexamethasone was offered and contributed to the tumour regression, but accelerated their growth thus a high dose of hydrocortisone was administered (>20 mg/m^2^/day) [48].

As mentioned, Ladjouze A. et al. identified 3/6 boys with TARTs, respective 3/8 females with adrenal masses (that required removal in two cases), in addition to one case of OART [38]. Yu L. et al., introduced the case of a Chinese boy who was admitted by the age of 14 y for a giant bilateral TARTs of 8.3 cm, respective 7.4 cm maximum diameter, that required surgical removal (after initial responsiveness to glucocorticoid replacement, TARTs enlarged because of non-compliance to therapy amid COVID-19 pandemic) [39].

### 2.7. Management

The mentioned studies included patients who were treated with glucocorticoid replacement therapy (hydrocortisone, dexamethasone, and prednisolone; hydrocortisone equivalent doses between 8 mg/m^2^/day and 100 mg/m^2^/day) in addition with mineralocorticoid replacement like fludrocortisone (doses between 0.05 mg/day and 0.25 mg/day) in SW forms [22,26,31,46,48,57]. One retrospective study on 16 individuals (mean age of 7.2 ± 6.4 y; female/male ratio of 10/6) from Lancaster, Pennsylvania showed that, despite high-dose of hydrocortisone, the abnormal hormonal assays are persistent, and that seems to be a distinctive feature of 3βHSD2 deficiency, a correction profile of hormones values more resistant to therapy compared to 21OH deficiency (persistent high ACTH was 10 times above normal, 17OH-Preg was 20 times as normal, and DHEA was 20 times as normal; in this study, the standard dose of hydrocortisone versus high-dose was 15.4 ± 4.9 mg/m^2^/day versus 37.8 ± 15.4 mg/m^2^/day, *p* < 0.0001) [22]. Additionally, some male patients received testosterone replacement therapy [26,31,45]. Urogenital anomalies were surgically corrected with hypospadias repair and orchidopexy [26,31,46,48,57]. Also, clitoral reduction was performed [47]. Tumours were surgically removed or followed up for a while depending on disease control methods [39,45,48,56].

### 2.8. Other Anomalies Reported in Patients with 3βHSD2 Deficiency

Two of the reported cases had 3βHSD2 deficiency and Klinefelter syndrome, which represents an interesting mix of androgen excess and hypogonadism (47,XXY/46,XX, respective 47,XXY) [48,51]. A patient with 3βHSD2 deficiency, coming from a non-consanguineous family, was displayed Bartter Syndrome due to a homozygous deletion from exon 1 to 19 of CLCNKB, in the context of uniparental isodisomy [41]. Another subject displayed a combination of autoimmune disorders, including Addison’s disease. She had a family history of CAH with SW, and was admitted for clitoral hypertrophy, hyponatremia, hyperkaliemia and elevated 17-ketosteroids. Later on, she developed premature ovarian insufficiency; vitamin B12 deficiency, and positive parietal cell autoantibodies. Computer tomography showed adrenocortical atrophy [43]. At this point, we do not have enough evidence to sustain a pathogenic connection between these disorders and 3βHSD2 deficiency, thus they seem incidental.

## 3. Discussion

The approach of heterogonous spectrum underling 3βHSD2 deficiency varies, the first challenge being its prompt recognition in severe cases with SW-associated acute presentation [70]. A multidisciplinary team is required to address various clinical manifestations and complications, from newborns to adults. Despite the fact that within the last decade the number of publications addressing this particular enzyme deficiency remained low, a level of awareness is mandatory. Nowadays, genetic assays are essential for an adequate diagnosis, thus, an index of suspicion concerning this particular type of CAH is necessary; yet there are still issues concerning the access to these investigations (for instance, some studies genetic analysis was not provided in a few mentioned papers).

Anomalies of virilization are reported in females and undervirilization in males. DHEA accumulation and conversion of androgens by normal 3βHSD type I lead to in utero virilization of females which includes clitoromegaly and labial fusion [32,48,57]. However, females rarely present with ambiguous genitalia in 3βHSD2 deficiency despite it generally being recognized that CAH is the main contributor of DSD in 46,XX individuals due to intra-uterine androgen exposure [24,71,72]. In case of this enzymatic deficiency, testosterone production by testicular Leydig cells is impaired, which leads to DSD. Undervirilization of males due to low testosterone and dihydrotestosterone production leads to hypospadias [57,64]. Also, gynecomastia in adult males may appear due to high levels of oestrogens, resulting from the conversion of testosterone and androstendione by the HSD17B5 and CYP19A1 enzymes [35].

Puberty onset might be affected in different types of CAH [73]. Premature pubarche described in some of the patients we mentioned may be caused by an elevation of testosterone and Δ4 steroids due to conversion by the 3βHSD type I enzyme, while type II enzyme is less productive [38].

Symptoms of non-classic forms of CAH (as seen in 21OH deficiency, 3βHSD2 deficiency or 11-hyroxylase deficiency) significantly overlap with POS, and many female patients actually remain undiagnosed regarding CAH criteria [74,75,76,77]. Both conditions lead to androgen excess, hirsutism, insulin resistance and anovulation (while androgen excess may be found in menopausal females with CAH, too) [74,75,76,78]. Considering the impact on a possible offspring, differential diagnosis between POS and non-classic CAH is essential [68,74]. While clinical manifestations may be similar, the clue in order to differentiate CAH from POS is to perform the panel of androgens assays [78,79]. A practical tool of differentiation remains 17OHP; however, baseline values may be normal in both CAH and POS, thus an ACTH stimulation test might be useful [80].

Notably, from an epidemiological perspective, the most common condition causing androgen excess is POS, affecting 10% of all females with reproductive age; while hirsutism relates to idiopathic forms followed by POS in 85% of cases [81]. The other conditions that represent 10–15% of disorders underling androgen excess are, apart from non-classical forms of CAH, Cushing syndrome, severe insulin resistance syndromes, and ovarian tumours with androgens overproduction [79,82,83]. The non-classical forms of CAH (regarding any type of enzyme defect, but, mostly, 21OH deficiency) represent 0.6 to 9% of all cases with androgen excess [84]. As an observation, we mention that reported POS elements in patients diagnosed with CAH do not represent an actual POS diagnosis, since positive diagnosis of POS means the exclusion of other causes underling androgens excess (as CAH) [38]. As of fertility/subfertility rates and pregnancy outcomes in females confirmed with 3βHSD2 deficiency, we found no particular study, as opposite to 21OH deficiency [85]. As a rule of thumb, females of reproductive age with non-classical CAH should be treated with minimum efficient doses of hydrocortisone, not dexamethasone, in order to restore menstrual cycle (for a limited period of time) and, in cases with severe hirsutism, oral contraceptives and/or antiandrogens like spironolactone are useful. The key of adjusting the regime is a patient—based approach following the continuously changing needs through life span [86,87].

In 3βHSD2 deficiency, adrenal hyperplasia is caused by hypersecretion of ACTH due to chronic cortisol deficiency [22]. The patients presented in one study were misdiagnosed as having 21OH deficiency and inadequately treated leading to large adrenal tumours thus surgery was necessary. This emphasizes the importance of providing an accurate diagnosis and management, including glucocorticoid substitution, guided by 17OH-Preg levels rather than 17OHP [38]. Adrenal glands enlargement should be differentiated by other causes of primary bilateral adrenal hyperplasia, either of macronodular or micronodular type, some of these entities also underling mutations like ARC5 tumour suppressor gene that is why genetic counselling might be helpful, as well [88,89,90,91]. A meta-analysis from 2020 pointed out the prevalence of adrenal masses in subjects diagnosed with CAH, considering both adrenal tumours (according to 6 cohort studies) and myelolipomas (based on 32 case reports) which is of 29.3%, respective 7.4%, with a median age at diagnosis of 36 y, and males being more affected than females [92]. Of course, the predominant form of CAH was 21OH deficiency, but generally adrenal imaging should be taken into consideration in other types of CAH too. This is especially the case under particular circumstances like poorly controlled disease, long standing medical history, abdominal pain, surveillance of prior diagnosed adrenal masses that are not primarily surgery candidates, etc. [92]. Of note, contemporary modern techniques of adrenal surgery allow synchronous bilateral laparoscopic adrenalectomy, but this should be restrained in individuals with CAH since glucocorticoid replacement might improve the imaging aspects of adrenal glands, as seen in TARTs [93,94,95,96]. On the other way, CAH might be listed among the many causes of adrenal incidentalomas. For example, a study on 637 patients diagnosed with adrenal incidentalomas (mean age of 62.7 ± 11.6 years) during an 8-year period of time showed a prevalence of 0.3% with respect to CAH [97].

Differential diagnosis of 3βHSD2 deficiency should be done with other forms of CAH, especially with the most frequent iteration—21OH deficiency [98,99]. Other conditions include androgen insensitivity and as mentioned POS [38,47]. Also, TARTs are taken into consideration in selective non-CAH cases [100]. Elevated levels of 17OHP may be associated with 3βHSD2 deficiency due to peripheral conversion by the 3βHSD type I enzyme. High 17OHP levels are false positives for 21OH deficiency in neonatal screening programs according to some of the mentioned studies [5,60,101,102,103,104]. Certain countries introduced screening protocols based on 17OHP immunoassays (dried blood spot) which are less useful in non-severe cases and non-classical forms of 21OH deficiency and non-21OH types [24,103,105]. Alternatively, within the first 24 h of life, 17OHP is falsely elevated in premature new-borns, in some cases of infants under physiological stress of birth, and, also, in 11-hydroxylase deficiency, and P450 oxidoreductase deficiency [106]. One useful tool for differential diagnosis is the assessment of 21-deoxycortisol, which has an elevated level only in 21OH deficiency [106].

A large panel of complications are reported for 3βHSD2 deficiency, starting from the disease outcome to iatrogenic issues. In terms of cardiovascular risk, it is assumed that the prevalence is similar to other forms of CAH due to obesity and diabetes mellitus often being caused by iatrogenic Cushing syndrome [35,45,48]. Generally, CAH associates a higher cardiovascular and metabolic risk, including in younger adults, under adrenocortical hormonal therapy, but the exact pathogenic loop is less understood [107]. Probably, androgen excess in females is a contributor to insulin resistance, which might be enhanced by glucocorticoids over-treatment [108]. However, a lifelong surveillance of these aspects is required, as part of a non-traditional clinical spectrum [107]. Long-term randomized trials are needed in order to highlight the cardio-metabolic risk and its age-dependent evolution in individuals with CAH, including 3βHSD2 deficiency [109].

Some patients may need higher doses of glucocorticoids than patients with 21OH deficiency in order to suppress the elevated androgens levels. In males with testosterone-responsive micropenis or who show delayed puberty, testosterone replacement therapy may be useful. One the other hand, high testosterone-related precocious puberty may be treated with gonadotrophin-releasing hormone analogues [110]. Urogenital surgery is performed to treat consequences such as undescended testis and hypospadias in males and genital virilization of females [35].

High levels of ACTH (thus suboptimal adrenal hormonal replacement) may lead to the development of TARTs and OARTs, but the exact prevalence is still unknown [35]. The mentioned 46,XY case reports show modified spermatogenesis (since CAH is listed among the non-obstructive caused of azoospermia), but also they indicate that TARTs might affect fertility in patients with 3βHSD2 deficiency [45,48,111]. Since first description (1940) of a TART in a 3-year-old boy who died of SW crisis, the scientific community has gathered an interesting amount of data; however, still, further longitudinal studies are mandatory in all types of CAH. In terms of treatment, there has been reported a tumour size reduction under high dose of glucocorticoids replacement which is the first step of their management [48,112]. A study of 8 cases with CAH-related TARSs also included a review of literature from 1990 to 2019 that analysed 23 articles. 123 underling subjects with a total of 223 CAH-associated TARTs were reviewed; the tumours profile showed 81% to be bilateral, and this aspect usually excludes malignancy (which is attributed to a solitary mass in most cases). Also, as pathogenic elements, some suggest the ACTH–tumour size correlation, whereas some suggest other growth factors. However, it is essential that, once the tumour is identified, a good hormonal control is established since 64% of tumours seemed reversible under adequate CAH management [113]. One study from 2021 showed that TARTs are less likely to be identified in non-classical forms of CAH (in this case—21OH deficiency); despite being recognized early in young boys (especially those with advanced skeletal age and increased 17OHP levels) TARTs have an increasing prevalence with age [114].

On the other hand, OART, generally recognized as an ovarian tumour in 46,XX females with CAH, has a rare occurrence, far less than TARTs in 46,XY males with CAH [115]. OART has been previously described in the literature in association with 3βHSD deficiency, and we mention a report from 1994 considering 3βHSD2 deficiency (although, over the years, there have been some terminology changes), as well as the recent study of Ladjouze A. et al. [38,116]. Generally, in patients with CAH, OARTs are contributors to gonadal dysfunction less than TARTs in males, in addition to contributing to poor disease control-induced hypogonadism in males, respective, associated androgen excess in females. The dysfunction of hypothalamus–pituitary–gonadal axis represents an indicator of CAH control; thus, in women it is important to continuously assess the menstrual cycle disturbances, while in men periodic testes ultrasound check-up is useful [5].

Overall, in older patients who went through newborn period and potential surgical corrections in early childhood, a significant medical and surgical burden is attributed to adrenal tumours (probably due to suboptimal hormonal replacement), a POS-like clinical panel starting from teenage years, and iatrogenic issues due to long time glucocorticoid exposure [38].

Of note, the modern society is oriented toward the improvement of life quality in addition to adequate case management, and recently a shift from evidence-based medicine to individualized (patient—oriented) medicine has been registered; this approach is particularly applicable in exceptional and challenging disorders as 3βHSD deficiency. CAH diagnosis may generate anxiety due to concerns about the risk of adrenal crises, DSD, and genital surgery as well as the long-term effects of hyperandrogenism in females in addition to fertility issues and genetic counselling concerning future offspring [24,117]. Multiple hospitalizations and medical admissions, and lifelong surveillance may deeply impact the quality of life.

Recently, the current COVID-19 pandemic revealed some categories of endocrine patients who are at higher risk of a severe coronavirus infection, and subjects with CAH-associated chronic adrenal insufficiency are no exception [118]. Moreover, as previously mentioned, non-adherence to hormonal replacement during pandemic era became an essential contributor to TARTs growth that might have led to surgical removal. [39].

Current limits of this topic come from its rarity and difficult diagnosis in both paediatric and adult population. Future perspectives include larger, multi-centric trials with a strong longitudinal component and hopefully enough data to obtain meta-analysis on CAHs, TARTs, OARTs, and overlapped phenotype with POS, fertility profile, as well as networking for excellence centres in gene sequencing.

## 4. Conclusions

To our knowledge, this is the largest study on published cases specifically addressing 3βHSD2 deficiency. We conclude that CAH due to 3βHSD2 deficiency represents an exceptional disease that may cause a wide spectrum of clinical presentations in relation with the severity of the enzyme deficit, a spectrum that many times is difficult to recognize and may remain under-diagnosed. In severe cases, newborns present adrenal crisis and genital ambiguity, while milder forms of the disease may be discovered later in life or misdiagnosed as POS. 3βHSD deficiency may be identified also among cases with an elevated 17OHP level at birth during screening for 21OH deficiency. Although exceptionally rare in general population, the disease seems to be more frequent in consanguineous families. Due to the possible impact in offspring regarding females with non-classical form, it is important to establish the differential diagnosis with POS. In order to address various complications like infertility, TARTs, adrenal tumours, cardio-metabolic risk, the management includes glucocorticoid and mineralocorticoid replacement, surgical correction of the urogenital anomalies, resection of tumours based on a case-by-case strategy and lifetime surveillance. An increased level of awareness is required.

## Figures and Tables

**Table 1:** Clinical spectrum of 3βHSD2 deficiency: original studies published on PubMed within the last decade (the papers are listed starting with the most recent, from 2022 to 2012) (References: [5,22,25,26,27,31,38,39,40,41,42,43,44,45,46,47,48,49,50,51,52,53,54,55,56,57,58,59,60,61]).

First Author/ Year of Publication/ Reference No.	Type of Study	Studied Population/ Number of Patients	Clinical Presentation	Therapy
Ladjouze A. 2022 [38]	Mixed single-centre, longitudinal and cross-sectional study Algeria Single centre	N = 14 patients (8 females, 6 males) of 10 families with 3βHSD deficiency	Females presented with: SW (5/8) SW + clitoromegaly (2/8) screening at birth (1/8) Males presented with: SW (1/6) genital anomalies (1/6) SW + genital anomalies (3/6) family screening (1/6) Virilization of girls: not significant (2/8) Prader stage 1 +clitoromegaly (2/8) Prader stage 2 + clitoromegaly and narrow opening of distal vagina) (4/8) Undermasculinization of boys: severely (2/6) and mildly (2/6) 4 boys underwent hypospadias correction. One boy had spontaneous puberty. 2 boys had premature pubarche Median IQ = 90 N = 3/14 with IQ < 70 (2/3 with a family history of unclassified global neuro-disability disorder 2 boys were diagnosed with TARTS following systematic testicular ultrasonography (one was inadequately treated). 3 out of 4 girls who reached menarche met the criteria for POS, while one had polycystic ovaries without prolonged amenorrhea or severe hirsutism. These 3 girls also presented with adrenal masses (adrenal cortical hyperplasia). One of them also presented with an ovarian mass (highly suggestive of OART).	Hydrocortisone: mean dose of 15.2 ± 0.8 mg/m^2^/day Fludrocortisone: 54 ± 25 μg/day (N = 11/14)
Li Z. 2021 [27]	Case report	1 male patient	9-y boy with enlargement of the testes for the last 5 y in addition to increased height growth (+1.36 SD for his age).	Hydrocortisone: 10–15 mg/m^2^/day, divided into 3–4 doses
Yu L. 2021 [39]	Case report	2 male siblings (46,XY)	2 brothers with hypospadias, orchialgia and testicular masses (TARTs) The younger sibling also presented tanned skin colour.	Hydrocortisone: 15–23 mg/m^2^/day + TARTs excision
Chen L. 2021 [31]	Case report	3 patients (2 males and 1 female)	Patient 1 (46,XY): embedded penis, hypospadias, dark-coloured scrotum, poor suction and regurgitation of milk, diarrhoea, irritability, convulsions, lack of sweating, short stature, intellectual and developmental delay Patient 2 (46,XY): short penis with hypospadias, scrotal skin pigmentation, poor suction, low weight gain, irritability, convulsions, lack of sweating Patient 3 (46,XX): skin pigmentation, pigmentation of the labia, slightly enlarged clitoris, intermittent vomiting, diarrhoea, malnutrition, developmental delay	The patient was receiving hormonal treatment at the time of the initial diagnosis. Patient 1: Hydrocortisone (at the age of 2–5 years the dose was 8–10 mg/m^2^/day). Fludrocortisone 4 long-acting testosterone intramuscular injections at a dose of 100 mg/m^2^ once every 15 days; topical application of dihydrotestosterone cream for 1 month. Patient 2: Hydrocortisone Fludrocortisone 6 intramuscular injections of gonadotropin (1500 U) one every 2 weeks long-acting testosterone injections (intramuscularly) in a dose of 100 mg/m^2^ once every 2 weeks hypospadias repair at the age of 2 years and 10 months Patient 3: Hydrocortisone Fludrocortisone No surgical correction was needed
Fanis P. 2020 [40]	Case report	1 male patient (46,XY)	At birth: ambiguous genitalia with scrotal hypospadias, bifid scrotum, and palpable gonads in the inguinal canal bilaterally At age of 15 days: SW crisis At age of 3.5 y: gynecomastia, adrenarche, SW crisis At age of 8.5: precocious puberty (a 4-y advance of bone age versus his biological age)	Hydrocortisone: 10 mg/m^2^/day Fludrocortisone: 100 μg/day
Guran T. 2020 [26]	Cross-sectional study	N = 31 patients (12 females, 19 males)	SH and non-SW forms certain levels of genotype–phenotype correlations	Hydrocortisone + Fludrocortisone in SW forms
Giri D. 2020 [41]	Case report	1 female (46,XX)	Weight loss, jaundice, poor feeding, hyponatremia, hypochloremia, hyperkalemia, metabolic alkalosis No ambiguity of the genitalia + Barter syndrome type 3	Oral sodium 16 mmol/kg/day, potassium supplements 6.5 mmol/kg/day Hydrocortisone 8 mg/m^2^/day Fludrocortisone 60 μg/day Indomethacin 9 mg 3 times daily
Reihani-Sabet F. 2020 [42]	Case report	1 case of preimplantation testing for 3βHSD deficiency	Preimplantation diagnosis followed by the identification of a healthy heterozygote and the birth of a healthy boy	-
Aslaksen S. 2019 [43]	Case report	1 patient with 3βHSD deficiency discovered during a whole-exome sequencing study of 142 Addison’s Disease patients	Hyperpigmentation of the genitalia, clitoris hypertrophy, hyponatremia and hyperkaliemia At 1 week of age: elevated 17-ketosteroids. The patient developed premature ovarian insufficiency; and vitamin B12 deficiency (The patient had a sister who died of adrenal crisis)	Hydrocortisone 20 mg/day Fludrocortisone 100 μg/day
Dundar A. 2019 [44]	Cross-sectional study	386 patients with CAH 6 patients with 3βHSD deficiency	SW + hyperpigmentation SW + vomiting, diarrhoea SW + ambiguous genitalia (male) SW SW Non-classic	Hydrocortisone + Fludrocortisone in SW forms
Lolis E. 2018 [45]	Case report	1 male (46,XY)	SW, penoscrotal hypospadias, micropenis, cryptorchidism, bifid scrotum TARTs	Hydrocortisone until the age of 15 → followed by Prednisolone (20–30 mg equivalent to hydrocortisone) Fludrocortisone: (highest dose of 250 μg/day) After bilateral orchiectomy at 33 y: testosterone replacement therapy when testosterone declined to 10 nmol/L accompanied by elevated gonadotropins
Donadille B. 2018 [25]	Case report	1 male (46,XY)	SW, micropenis, intrascrotal testes, perineal hypospadias Normal pubertal development since the age of 15 y At 24 y: normal spermogram except for suboptimal sperm vitality with normal sperm count and percentage with typical morphology	Hydrocortisone: 17.3 mg/m^2^/day Fludrocortisone: 75 μg/day
Shehab MA. 2018 [46]	Case report	1 phenotipic male patient with mosaic Klinefelter syndrome and CAH (47,XXY/46,XX)	Penoscrotal hypospadias, bilateral cryptorchidism, accelerated growth with appearance of axillary and pubic hair with progressive blackening of skin, rapid phallic enlargement, he later developed gynecomastia + Klinefelter syndrome	Hydrocortisone 5 mg in the morning and 10 mg at night Fluodrocortisone 0.1 mg Orchidopexy right testis, orchiectomy left testicle
Teasdale SL. 2017 [47]	Case report	1 patient with 46,XY (assigned as female)	At birth: ambiguous genitalia: testes in scrotum, vaginal introitus, perineal urethra, small phallus (no SW) Initially misdiagnosed as androgen insensitivity, however hCG test was not consistent with androgen insensitivity → diagnosis established after adrenarche	Gonadectomy Elective clitoral reduction, Estrogen hormone replacement therapy. The patient did not require glucocorticoid replacement
Güven A. 2017 [48]	Case report	2 male siblings (46,XY)	Case 1 (age of 3.5 y): hypospadias, scrotum bifidum, left cryptorchidism, adrenal insufficiency + TARTs Case 2 (age of 22 months): hypospadias, scrotum bifidum + TARTs	Case 1: Hydrocortisone (13–100 mg/m^2^/day), fludrocortisone (0.1 mg × 2/day) Surgical correction of hypospadias and left orchidopexy Biopsy to rule out Leydig cell tumor Case 2: Hydrocortisone (15–25 mg/m^2^/day) Hypospadias surgical correction + Of note: Case 1: increased plasma renin activity in spite of strict fludrocortisone treatment Case 2: patient could not maintain dexamethasone treatment due to becoming obese (the treatment was switched back to high-dose hydrocortisone) in order to control TARTs
Panzer K. 2017 [49]	Case report	1 male (46,XY)	Hypotonia, non-specific dysmorphic facial findings (frontal bossing, hypotelorism, low nasal bridge, anteverted nares), perineal hypospadias, bifid scrotum, penile chordee, descended testes bilaterally	Steroids and electrolyte replacement, surgical correction of urogenital anomalies
Scaramuzzo RT. 2017 [50]	Case report	2 sisters (46,XX)	SW Normal genitalia Failure to thrive, severe dehydration, hyponatremia, hyperkaliemia, adrenal insufficiency, adrenal hyperplasy	Sister 1: Hydrocortisone 19 mg/m^2^/day + fludrocortisone 0.05 mg/day Sister 2: Hydrocortisone 26.5 mg/m^2^/day + Fludrocortisone 0.15 mg/day in 2 doses
Gortakowski M. 2016 [51]	Case report	1 patient (47,XXY)	Association with Klinefelter syndrome Short stature, premature pubarche, facial acne, pubarche at the age of 4 y, prepubertal testes, bone age higher than chronological age	Therapy with growth hormone, aromatase inhibitor
Levy-Shraga Y. 2016 [52]	Case report	1 female (46,XX)	No evidence of androgen excess (diagnosed through screening for 21OHD deficiency)	Initially: hydrocortisone 20 mg/m^2^/day + fludrocortisone 0.2 mg/day At the age of 5 y: hydrocortisone 9 mg/m^2^/day + fludrocortisone 0.1 mg/day
Bizzarri C 2016 [53]	Case report	1 male (46,XY)	Perineal hypospadias, palpable testes within the labial scrotal folds, microphallus Recurrent vomiting, hypoglycemia and hyponatremia	Hydrocortisone 30 mg/m^2^/day Fludrocortisone 0.05 mg/day NaCl 6 mEq orally 6 times a day
Probst-Scheidegger U. 2016 [54]	Case report	1 female (46,XX)	Diagnosed at neonatal screening SW No DSD	Hydrocortisone + Fludrocortisone replacement
Konar MC. 2015 [55]	Case report	1 female patient with suspected 3βHSD deficiency	Recurrent hypoglycaemia, adrenal insufficiency (in vitro fertilisation, no consanguinity, no gene testing)	NA
Vukina J. 2015 [56]	Case report	1 male patient	The patient presented with testicular pain and swelling The patient had a history of hypospadias, bilateral cryptorchidism, early growth spurt, frequent asthma flairs, salt craving, and a grandfather with precocious puberty Bilateral TARTs Azoospermia (no gene testing)	Orchiectomy
Burckhardt MA. 2015 [57]	Case report	1 patient (46,XY) male	Perinatal asphyxia, severe hypospadias, cryptorchidism, undervirilization Neonatal hypoglycemia, hyponatremia, hyperkalemia At puberty: virilization, palpable gonads in the inguinal area, gynecomastia Histology of the testes: no evidence of malignancy risk (no persisting immature foetal gonocytes, no premalignant germ cells), Sertoli-cell-only pattern Neurological impairments due to perinatal asphyxia	Replacement therapy with fludrocortisone and hydrocortisone At age of 15.5 y: orchidopexy
Benkert AR. 2015 [22]	Retrospective study	16 patients	Positive newborn screening, with 17OHP from filter paper (N = 11) Perinatal SW adrenal crisis (N = 1) High-risk molecular screening (N = 2) Hypospadias requiring surgical correction (all 6/16 males) No genital ambiguity in females (10/16) Complications: Adrenal insufficiency: adrenal crisis (SD N = 2, HD N = 4), hypoglycemia (SD N = 1, HD N = 2), hyperkalemic acidosis (SD N = 4, HD N = 2) Failure to thrive (SD N = 4, HD N = 1) Hypospadias (all 6 male patients) Advanced skeletal maturation (HD N = 4) Hirsutism, acne (HD N = 5) TARTs (HD N = 2) POS (HD N = 2) Arterial hypertension (SD N = 7, HD N = 3) Obesity (HD N = 5) Cushing syndrome (SD N = 3, HD N = 5) Delayed skeletal maturation (SD N = 1, HD N = 1)	SD: dexamethasone 0.22 ± 0.07 mg/m^2^/day HD: dexamethasone 0.54 ± 0.22 mg/m^2^/day
Baquedano MS. 2015 [61]	Case report	1 female (46,XX)	Precocious puberty at age of 7 months Clitoromegaly (non-SW)	No need for mineralocorticoid replacement
Araújo VG. 2014 [58]	Case report	1 male (46,XY)	Diagnosed during new-born screening for CAH 2.5 cm phallus, penoscrotal hypospadias, incompletely fused labioscrotal folds, palpable gonads bilaterally, vomiting, dehydration, hyponatremia, hypercalcemia	Glucocorticoid + mineralocorticoid replacement
Takasawa K. 2014 [59]	Case report	1 female (46,XX)	Labia minora fusion, clitoromegaly, Prader stage II, skin pigmentation, weight loss	Glucocorticoid + mineralocorticoid replacement
Jeandron DD. 2012 [60]	Case report	1 female (46,XX)	At age of 13 y: SW crisis (no SDS)	Glucocorticoid + mineralocorticoid replacement
Claahsen-van der Grinten HL. 2012 [5]	Case report	1 male (46,XY)	TARTs with extra-testes involvement	Surgery for perirenal mass

Abbreviations: 3βHSD deficiency = = 3-beta-hydroxysteroid dehydrogenase; 21OHD = 21-hydroxylase deficiency; CAH = congenital adrenal hyperplasia; IQ = intelligence quotient; N = number of patients; OART = ovarian adrenal rest tumour; SW = salt wasting; TARTs = testicular adrenal rest tumours; Y = years.

**Table 2:** Hormonal panel in patients diagnosed with 3βHSD2 deficiency: studies published within between 2022 and 2012 (References: [22,25,26,27,31,38,39,40,41,43,45,46,47,48,49,50,51,52,53,54,55,56,57,58,59]).

First Author/ Year of Publication/ Reference No.	Hormonal Panel
Ladjouze A. 2022 [38]	17OHP: above upper limit (N = 12/14; the other two patients were under treatment with hydrocortisone) Median (range) = 73.7 (0.37–164.3) nmol/L 17OH-Preg: above upper limit (N = 13/14) Median (range) = 139.7 (10.9–1500) nmol/L (a value > 90 nmol/L is sugestive the diagnosis of 3βHSD2 deficiency) ACTH: above upper limit (N = 4/14) DHEA: above upper limit (N = 8/14) within normal range (N = 4/14) below normal range (N = 2/14 under treatment with daily hydrocortisone) Androstenedione: above upper limit (N = 3/14) within normal range (N = 6/14) below lower limit (N = 1/14) DHEA-S: Median (range) = 501.2(9.4–5441.3) nmol/L
Li Z. 2021 [27]	High levels of 17OHP and testosterone Very high values of DHEAS and androstenedione
Yu L. 2021 [39]	17OHP: elevated (values between 20–60 nmol/L) ACTH: elevated (values between 500–1500 pg/mL; cortisol with values between 100–200 nmol/L) Testosterone: elevated (values around 20 mol/L) Following surgery, hormone levels were: ACTH = 4.090 pg/mL Plasma cortisol = 0.000 μg/dL Testosterone = 7.54 nmol/L 17OHP = 0.47 nmol/L
Chen L. 2021 [31]	Patient 1: 17OHP >75.75 mmol/L (normal: <29.1 mmol/L) ACTH = 34 pg/mL (normal: <46 pg/mL) Testosterone = 4.02 nmol/L (normal: <0.69 nmol/L) DHEA = 5.92 μmol/L Androstenedione >35 nmol/L Patient 2: 17OHP = 3.7 mmol/L ACTH = 262 pg/mL Plasma cortisol = 810 nmol/L (normal: 124–662 nmol/L) Testosterone = 4.3 nmol/L (normal: <0.69 nmol/L) DHEA < 0.41 μmol/L (normal: <0.41) Androstenedione = 2.13 nmol/L Patient 3: 7OHP > 75.5 mmol/L ACTH > 1250 pg/mL Cortisol = 497 (normal: 124–662 nmol/L) Testosterone = 2.13 nmol/L (normal: <0.69 nmol/L) DHEA > 27.1 μmol/L (normal: <0.41) Androstenedione > 35 nmol/L
Fanis P. 2020 [40]	At age of 8.5 y: 17OHP = 394 ng/mL (normal: <6.3) ACTH = 3312.8 pg/mL (normal: 10–60) Plasma cortisol = 0.7 μg/dL (normal: 6–19) Testosterone = 738 ng/dL (normal: 115–403) DHEA = 633 ng/mL (normal: 0.46–3.22) Progesterone = 7 ng/mL (normal: <0.5 ng/mL) Plasma Renin Activity = 116.86 ng/mL/h (normal: 1.4–7.8 ng/mL/h)
Guran T. 2020 [26]	Median values (N = 31, mean age of 6.6 ± 5.1 y): 17OHP = 2.38 nmol/L (normal: 0.27–7.73) 17OH-Preg = 83.3 nmol/L (normal: 45.4–125) Plasma cortisol = 21.1 nmol/L (normal: 1.78–113) Testosterone = 0.55 nmol/L (normal: 0.17–1.94) DHEA = 39.9 nmol/L (normal: 8.01–105) Androstenedione = 0.97 nmol/L (normal: 0.27–2.82) 11-deoxycortisol = 1.09 nmol/L (normal: 0.37–2.85)
Giri D. 2020 [41]	17OHP >110 nmol/L DHEA-S = 7 μmol/L (normal: 0.9–11.6)
Aslaksen S. 2019 [43]	Elevated 17-ketosteroids (at one week of age) After the age of 50 y: all mineralocorticoids, and most glucocorticoids and androgens were below the detection limit, except for: Tetrahydrocortisol = 0.308 nmol/L 5α-tetrahydrocortisol = 0.187 nmol/L Testosterone = 0.066 nmol/L DHEA-S below the normal range. ACTH highest levels = 130 pmol/L (normal: 2.0–11.6)
Shehab MA. 2018 [46]	17OHP = 2.15 ng/mL (normal: 0.5–2.1) 17OH-Preg = 2097 ng/dL (normal: <72) ACTH = 269 pg/mL (normal: 0–46) Plasma cortisol = 129 nmol/L (normal: 138–690) Testosterone = 276 ng/dL (normal: <42) DHEA-S = 331.8 (normal: <186) Androstenedione = 34 ng/dL (normal: 6–115)
Lolis E. 2018 [45]	17OHP = 15 nmol/L (normal: <5) Testosterone = 30 nmol/L (normal: 10–30), with later decline to 10 nmol/L accompanied by elevated gonadotropins (bilateral orchiectomy) DHEA = high levels Androstenedione = 12 nmol/L (normal: 1.2–5.0) Estradiol = 164 pmol/L (normal: <130) Renin = highest level of 442 mIU/L (normal: 2.4 to 41)
Donadille B. 2018 [25]	At age of 12 days: 17OHP = normal 17OH-Preg = elevated At age of 22 y: 17OHP = 0.022 nmol/L (normal: 1.2–7.6) 17OH-Preg = 4.38 nmol/L (normal: <6) Plasma cortisol = 0.039 nmol/L (normal: 212–607) Testosterone = 13.82 nmol/L (normal: 9–38) DHEA = 6.48 nmol/L (normal: 3.5–52) Androstenedione = 1.46 nmol/L (normal: 0.9–7) 11-deoxycortisol = 0.006 nmol/L (normal: <3) Aldosterone = 0.011 nmol/L (normal: 0.4) FSH = 3.6 UI/L (normal: 1.4–18.1) LH = 4.8 UI/L (normal: 1.5–9.3)
Teasdale SL. 2017 [47]	17OH-Preg = 376.7 nmol/L (normal: 0.0–10.0) ACTH = 147 ng/L (normal: 9–51) Plasma cortisol = 524 nmol/L Testosterone = 2.2 nmol/L (normal: 0.3–2.8) DHEA = 9.1 μmol/L (normal: 0.3–1.6) Androstenedione = 4.5 nmol/L (normal: 1.0–1.8) Neonatal hCG stimulation test: 4–5 days: free testosterone = 4.8 pmol/L (day 0) (normal: <2.0 pmol/L), DHT = 1.54 nmol/L (day 1), LH = 8 U/L (day 1), FSH < 2 U/L (day 1) 9–10 days: testosterone = 3.9 pmol/L (day 5) (2–4 × baseline), DHT = 1.4 nmol/L (day 5) (T:DHT < 10:1), LH = 2 U/L (day 5), FSH < 2 U/L (day 5)
Güven A. 2017 [48]	Case 1: 17OHP = 29 ng/mL ACTH = 546 pg/mL DHEA-S = 1550 μg/dL Case 2: ACTH = 161 pg/mL (normal: 6–46) ACTH stimulation test: Case 1 at 0′: ACTH >1250 pg/mL, Cortisol = 0.45 μg/dL, 17OHP = 27.5 ng/mL, Androstenedione = 0.2 ng/mL, Testosterone = 0.18 ng/mL, Progesterone = 0.6 ng/mL, DHEA-S = 1550 μg/dL Case 1 at 30′: Cortisol = 0.73 μg/dL, 17OHP = 35.1 ng/mL, Androstenedione = 0.4 ng/mL, Testosterone = 0.18 ng/mL, Progesterone = 0.6 ng/mL Case 2 at 0′: ACTH = 507 pg/mL, Cortisol = 16.5 μg/dL, 17OHP = 112 ng/mL, Androstenedione = 16.7 ng/mL, Testosterone = 4.5 ng/mL, Progesterone = 36.1 ng/mL, DHEA-S = 3870 μg/dL Case 2 at 30′: Cortisol = 17.6 μg/dL, 17OHP = 106 ng/mL, Androstenedione = 14.2 ng/mL, Testosterone = 4.4 ng/mL, Progesterone = 43.3 ng/mL, DHEA-S = 2985 μg/dL
Panzer K. 2017 [49]	17OHP = 16.9 nmol/L (normal: 1.3–6.4) 17OH-Preg = 119.0 nmol/L (normal: 0.3–26.2) ACTH = 8.4 pmol/L (normal: 1.3–10.6) Plasma cortisol = 1462.3 nmol/L (normal: 77.3–303.5) Testosterone = 1.6 nmol/L (normal: 0.7–1.7) DHEA = 95.4 nmol/L (normal: 1.7–26.4) Androstenedione = 9.7 nmol/L (normal: <1.8) 11-deoxycortisol = 9.9 nmol/L (normal: <5.9) 11-desoxycorticosterone = 0.2 nmol/L (normal: 1–2.07)
Scaramuzzo RT. 2017 [50]	Sister 1: 17OHP = 259 ng/mL (high) Testosterone = 0.351 ng/mL (high) Androstenedione > 12 ng/mL Sister 2: 17OHP = 314 ng/mL ACTH = 0.896 ng/mL DHEA = 730 ng/mL
Gortakowski M. 2016 [51]	17OHP = 42 ng/dL (normal: <116 ng/dL) Testosterone = 43 ng/dL (normal: <3–10 ng/dL) DHEA-S = 365 μg/dL (normal: 42–109 μg/dL) LH = 0.2 mIU/mL TSH, free T4, anti-tissue transglutaminase antibody, and IGF1 and IGFBP3 within normal range After ACTH stimulation test: 1 h after iv cosyntropin 250 μg: 17OH-Preg = 1979 ng/dL (normal: 88–675) 17OH-Preg/17OHProg = 35.3 (normal: 0.5–6.3) Plasma cortisol = 36.7 μg/dL (normal: 15–36) 17OH-Preg(nmol/L)/cortisol (μmol/L) = 53.9 (normal)
Levy-Shraga Y. 2016 [52]	17OHP = 500 nmol/L, at 6 days 181 nmol/L (normal: <7.5 nmol/L) Plasma cortisol = 292 nmol/L (normal: 138–690) Testosterone >55 nmol/L (normal: 0.7–2.2) Androstendion >34.5 nmol/L (normal: 0.7–10.5) 11-deoxycortisol = 143.2 nmol/L (normal: <23) Plasma renin activity >50 ng/mL/h (normal: 8–17)
Bizzarri C 2016 [53]	17OHP = 9620 ng/dL (normal: 7–77) ACTH = 577 pg/mL (normal: 96–135 pg/mL) Plasma cortisol = 10.78 μg/dL (normal: 0.6–19.8) Testosterone = 1320 ng/dL (normal: 1–177) DHEA-S = 473.1 μg/dL (normal: 91–376) Androstenedione = 1990 ng/dL (normal: 5–45) Renin: elevated aldosterone and renin
Probst-Scheidegger U. 2016 [54]	17OHP = 124 nmol/L (increased) ACTH = 549 ng/L (increased) Cortisol = 92 nmol/L (decreased) DHEA-S = 12 micromol/L (normal) 11-deoxycortisol = 54 nmol/L (normal)
Konar MC. 2015 [55]	17OHP = 11.8 ng/mL (normal: 0.2–2.00) ACTH = 183.9 pg/mL (normal: 7.2–63.3) Plasma cortisol = 2.4 μg/dL (normal: 3.7–19.4) Testosterone = 71.7 ng/dL (normal: 10–25) DHEA = DHEA-S = 191.8 μg/dL (normal: 3.4–123.6)
Vukina J. 2015 [56]	High 17OHP to 17OH-Preg ratio ACTH = 3050 pg/mL Plasma cortisol = 0.3 μg/dL (normal: 4.5–22.7) DHEA-S = 145 μg/dL (normal: 65–334)
Burckhardt MA. 2015 [57]	17OHP = 967 nmol/L (normal: <30 nmol/L) ACTH = 2638 (normal: 9.0–50) ng/L Plasma cortisol = 284 (normal: 140–470) nmol/L Testosterone = 171 pmol/L (normal: 6.4–27) DHEA >105 (normal: <3) nmol/L Androstenedione >34.5 nmol/L
Benkert AR. 2015 [22]	17OH-Preg: mean SD = 370 ng/dL, mean HD = 5858 ng/dL ACTH: mean SD = 100 pg/mL, mean HD = 817 pg/mL DHEA: mean SD = 71 ng/dL, mean HD = 1292 ng/dL Under HD/SD: ACTH, 17OH-Preg and DHEA were elevated relative to control siblings, mainly in the HD group. Early morning glucose levels were 8% lower in patients compared to control siblings. Serum osteocalcin level were low in patients, especially in HD group.
Araújo VG. 2014 [58]	17OHP=5170 ng/dL (normal: <2000), 2430 ng/dL (normal: <200) 17OH-Preg = 1080 ng/dL (normal: <10) ACTH = 50.18 pg/mL (normal: 0–46) Cortisol = 5.29 μg/dL (normal: 5–25) Androstenedione>1000 ng/dL (normal: 90–460) 17OH-Preg/cortisol = 224 (proposed criteria > 94 (45))
Takasawa K. 2014 [59]	17OHP = 66.6 nmol/L (normal: 2.94) 17OH-Preg = 909 nmol/L (normal: 12.6) ACTH = 32.5 pmol/L (normal: 0.3–2.9) Cortisol = 0.193 (normal: 0.3) DHEA = 262 nmol/L (normal: 10.3) Androstenedione = 3.91 (normal: 1.37) urinary pregnanendiol, urinary pregnanentriol, urinary androstenedione elevate

Abreviations: ACTH = adrenocorticotropic hormone; 17OH-Preg = 17-hydroxypregnenolone; 17OHP = 17-hydroxyprogesterone; 3βHSD deficiency = 3-beta-hydroxysteroid dehydrogenase; 21OHD = 21-hydroxylase deficiency; CAH = congenital adrenal hyperplasia; DHEA-S = dehydroepiandrosterone sulfate; DHEA = dehydroepiandrosterone; hCG = Human Chorionic Gonadotropin; HD = high dose; IQ = intelligence quotient; FSH=Follicle-Stimulating Hormone; LH = Luteinizing Hormone; N = number of patients; OART=ovarian adrenal rest tumour; SW = salt wasting; SD = standard dose; TARTs = testicular adrenal rest tumours; Y = years.

**Table 3:** Genetic testing in patients diagnosed with 3βHSD2 deficiency, according to original studies/case reports published on PubMed within the last decade (starting from 2022) (References: [5,22,25,26,27,31,38,39,40,41,43,44,45,46,47,48,49,50,52,53,54,57,58,59,60,61]).

First Author Year of Publication/ Reference No.	Genetic Testing of *HSD3B2* Gene
Ladjouze A. 2022 [38]	N = 12/14: homozygous, null mutation, c.665C > A (p.Pro222Gln) N = 2/14 (sisters): homozygous, c.453_464del (p.Thr152_Pro155del)—novel mutation 8 out of the 10 Algerian families were consanguineous (the parents were first-degree cousins in 4 families, and second-degree cousins in 4 families)
Li Z. 2021 [27]	c.674 T > A (p.V225D) (maternal and paternal inheritance) (China)
Yu L. 2021 [39]	c.674T > A (maternal inheritance) (China) c.776 C > T (paternal inheritance)
Chen L. 2021 [31]	Patient 1: frameshift mutation, c.154_162delinsTCCTGTT, exon 3 (novel mutation) nonsense mutation, c.1003C > T, exon 4 Patient 2: missense mutation, c.424G > A, exon 4 missense mutation, c.674T > A, exon 4 (novel mutation) Patient 3: missense mutation, c.776C > T, exon 4 nonsense mutation, c.1003C > T, exon 4
Fanis P. 2020 [40]	novel nonsense mutation—p.Lys36Ter (homozygosity, consanguineous parents) in addition to a heterozygous status for *CYP21A2* gene—p.Val281Leu
Guran T. 2020 [26]	frameshift, c0.271_275delCA (p.H92Qfs*32) frameshift, c. 959_960insC (p.L321Ifs*4) frameshift, c0.934delC (p.F314Sfs*54) frameshift, c0.429_430insAA (p.E144Kfs*31) missense, c0.320T > A (p.L107Q) missense, c0.1076T > C (p.L359P) missense, c0.1063T > C (p.W355R) missense, c0.967A > G (p.N323D) missense, c0.911T > C (p.L304P) missense, c0.733G > C (p.A245P) missense, c0.652T > C (p.S218P) (Turkey, 6 novel mutations)
Giri D. 2020 [41]	homozygous, nonsense, c.745C > T (p.Arg249*) Association of Barter syndrome type 3 due to homozygous deletion from exon 1 to 19 of CLCNKB) and 3βHSD deficiency, in the context of uniparental isodisomy (non-consanguineous parents, modified gene present only in mother)
Aslaksen S. 2019 [43]	homozygous, nonsense, c.15C > A (p.Cys5Ter), exon 2
Dundar A. 2019 [44]	homozygous, c.142 + 1G > T, intron 2 homozygous, p.N323D, exon 4 homozygous, p.V127E, exon 4 homozygous, p.S218P, exon 4 homozygous, p.R335X, exon 4 heterozygous, p.L304P, exon 4 (Anatolia, 4 novel mutations)
Shehab MA. 2018 [46]	47,XXY/46,XX homozygous miss-sense × 2 heterozygous V299I (GTA > ATA), S309T (TCC > ACC), Q311R (CAA > CGA) consanguineous
Lolis E. 2018 [45]	Cys-72-Arg in homozygote form or as compound heterozygous with deletion
Donadille B. 2018 [25]	homozygous, loss of function, 687del27, exon4 (consanguineous)
Teasdale SL. 2017 [47]	novel mutation—931C > T(p.Gln311) variant in addition to prior described c.244G > A (p.Ala82Thr).
Güven A. 2017 [48]	novel mutation—homozygous, p.W355R (c.763 T > C), exon 4 consanguinity (parents were second-degree cousins)
Panzer K. 2017 [49]	complete uniparental isodisomy of chromosome 1 homozygous, missense c.424G > A (p.E142K)
Scaramuzzo RT. 2017 [50]	homozygous, missense, c.969T > G (p.N323K) homozygous, missense, c.969T > G (p.N323K) (Morocco) consanguinity
Levy-Shraga Y. 2016 [52]	homozygous, missense, c.c664a C > A, exon 4 consanguinity (parents were second-degree cousins)
Bizzarri C 2016 [53]	novel homozygous frameshift mutation—a single nucleotide deletion at codon 319 (GTC(Val)x2192;GC) first mutation in Italy non-consanguineous
Probst-Scheidegger U. 2016 [54]	novel mutation nonsense, c.503delC nonsense, c.512G > A (no consanguinity)
Burckhardt MA. 2015 [57]	homozygous, loss of function, c.687del27, exon 4 consanguinity
Benkert AR. 2015 [22]	homozygous c.35G > A
Baquedano MS. 2015 [61]	novel missense mutation homozygous, pG250V
Araújo VG. 2014 [58]	homozygous, missense, c.665C > A, exon4 consanguinity
Takasawa K. 2014 [59]	missense, c.A569G missense, c.T652C (2 novel mutations)
Jeandron DD. 2012 [60]	novel nonsense homozygous mutation Q334X
Claahsen-van der Grinten HL. 2012 [5]	homozygous 694C > G

## Data Availability

Not applicable.

## Abbreviations

ACTH	Adrenocorticotropic Hormone
3βHSD	3beta-hydroxysteroid dehydrogenase
CAH	congenital adrenal hyperplasia
CRF	Corticotrophin Releasing Factor
DHEA-S	dehydroepiandrosterone sulfate
DHEA	dehydroepiandrosterone
DSD	disorders of sex development
LC-MS/MS	Liquid chromatography tandem–mass spectrometry
IQ	intelligence quotient
OART	ovarian adrenal rest tumours
Preg	pregnenolone
POS	polycystic ovaries syndrome
17OHP	17-hydroxyprogesterone
17OH-Preg	17-hydroxypregnenolone
21OHD	21-hydroxylase
TARTs	testicular adrenal rest tumours
SW	salt wasting
